# Supplementary material for: One million quality factor integrated ring resonators in the mid-infrared
Source: Nanophotonics. 2025 Mar 24;14(7):1009–15. doi: 10.1515/nanoph-2024-0761 (PMC11980867; doi:10.1515/nanoph-2024-0761)
Supplement: Supplementary file 1 — Supplementary Material Details [file j_nanoph-2024-0761_suppl_001.pdf]

Supplementary Material

Measurement Method

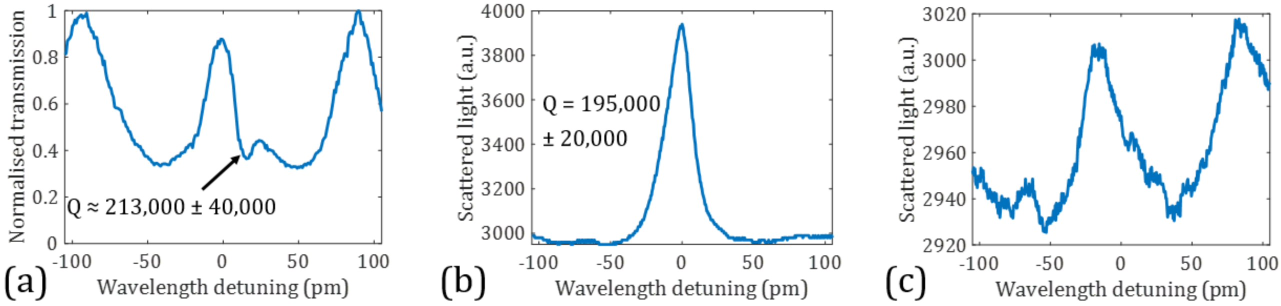

Fig. S5: Validation of our measurement method in scattering mode for a sample ring (for a ring resonator with a 250 μm radius and a gap of 250 nm). (a) Usual measurement in transmission mode, where the resonance is obscured by the Fabry-Perot resonances of the chip, but still with a detectable Q of around 213,000. (b) Resonance obtained in scattering mode, where the measured Q is 195,000 and hence very close and consistent with the transmission measurement. The Fabry-Perot resonances are almost not visible at all and hence not an issue, contrary to the transmission measurement. (c) Scattered light from the bus waveguide, where the Fabry-Perot resonances can be seen.

In the following, we discuss the measurement method in more detail.

First, we provide a comparison with the usual transmission measurement to validate our method in scattering mode with the top view MIR camera. Fig. S5 provides an example with a direct comparison between a resonance measured in transmission mode (Fig. S5a) and scattering mode (Fig. S5b). We used a resonator which showed a lower Q_{in}, but an increased coupling strength (radius 250 μm, waveguide width 3.25 μm, gap 250 nm), such that we can also observe the resonance in transmission. The ring is still undercoupled ($Q_c/Q_{in} \approx 18$), but the resonance is now clearly visible. This example also highlights the benefits of our new scattering technique. In particular, in transmission, the resonance is superimposed with the Fabry-Perot resonances formed by the chip end-facets, with the latter strongly hiding the signature of the ring resonance which exhibits a poor contrast. Although the resonance Q factor can still be extracted (Q around 213,000 ± 40,000), additional signal processing might be required to increase its visibility. On the other hand, in scattering mode, we obtain the Q-factor value more reliably, without the need for additional analysis. The result of 195,000 ± 20,000 obtained matches very well with that retrieved in the transmission mode. The error bars of the scattering mode are discussed in the main text, while the error bars for the transmission mode are dominated by the uncertainty in separating it from the Fabry-Perot resonances.

Second, we would like to point out that while we cannot directly measure the extinction ratio in the scattering mode, we can infer a direct estimation of the field enhancement (FE) factor afforded by the ring resonance, which can be further used to estimate the extinction ratio. The ability to directly measure the FE factor is also a strong point of the method, as usually this is only indirectly inferred from the extinction ratio. We illustrate this FE estimation for the example of the one million Q resonance (from Fig. 3a). As we are strongly under-coupled, we only expect a weak field enhancement. The analysis is illustrated in Fig. S6. We first select two areas with the exact same pixel size to record the scattering from the ring and from the bus waveguide as a function of time (i.e. laser spectral detuning). Within the area located on the ring, the scattering intensity on resonance (red line) can be measured as well as the background signal off resonance. Indeed, some roughly constant background light reaches the camera, creating an offset of the measured signal from the ring as well as from the waveguide areas. Within the second area centered around the bus waveguide (same waveguide length as that of the ring area), we record the scattering from the bus waveguide for the same spectral detuning of the input laser (blue curve). As in this case the ring is strongly under-coupled, the change of the power in the bus waveguide on

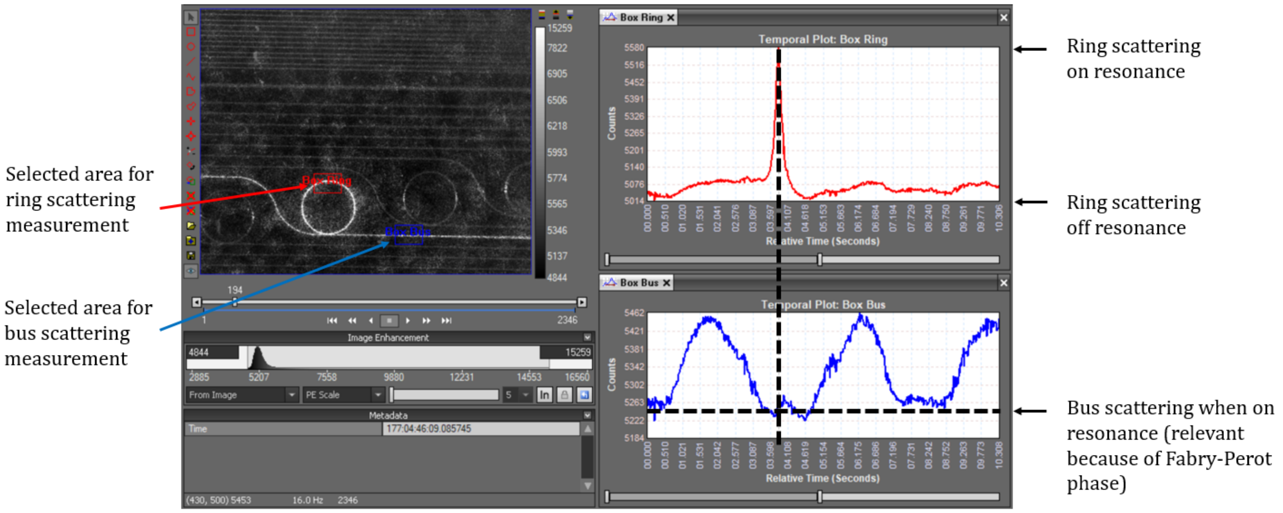

Fig. S6: Illustration of the FE factor extraction for the example of a one million Q resonance corresponding to Fig. 3a (ring radius 250 μm, waveguide width 5 μm, gap 500 nm). The wavelength tuning range that corresponds to the show time axis over 10 s is 210 pm. The counts correspond to the average count value over the selected area.

resonance is negligible compared to off resonance. Instead, this signal shows power oscillations that originate from the Fabry-Perot resonances from the chip end-facets (which were also visible in the direct transmission signal). The background offset for this area is estimated to be the same as for the ring area. In this particular case, the scattered light from the ring on resonance gives a count value of 5580, while the scattered light from the bus waveguide is only 5250, both above the background offset (measured off-resonance) of 5014. From these, we infer a FE factor of (5580 - 5014) / (5250 - 5014) = 2.4. From this we can calculate the corresponding extinction ratio using

$$|FE|^2 = \frac{P_{cavity}}{P_{bus}} = \frac{K/Q_{in}}{(\omega-\omega_0)^2 + \left(\frac{1+K}{Q_{in}}\right)^2 \frac{\omega_0^2}{4}} \frac{\omega_0 c}{n_g L} \overset{\omega=\omega_0}{=} \frac{2Q_{in}\lambda}{\pi n_g L} \frac{K}{(1+K)^2} \tag{2}$$

(see Ref. [1] for example) and

$$ER = \frac{1}{T} = \left(\frac{1+K}{1-K}\right)^2 \tag{3}$$

where $K = Q_{in}/Q_c$. This gives ER = 0.09 dB for our ring resonator. This is consistent with the fact that we do not detect any resonance in transmission. It is interesting to note that the depth in transmission 1 - T follows the same behavior as the field enhancement relative to K:

$$1 - T = 4\frac{K}{(1+K)^2} \tag{4}$$

The amplitude of the field enhancement scales linearly with Q_{in} where the transmission depth is just dependent on K. The limit $|FE|^2 = 1$ is achieved for an intrinsic quality factor $Q_{in}^{lim} = 2\frac{\pi n_g L}{\lambda} \approx 10,000$ and this limit leads to $|FE(Q_{in}^{lim})|^2 \approx 1-T$. For larger intrinsic quality factors, the field enhancement method is able to characterize a large value K (or $1/K$) easier than the transmission method. Our measurement method is therefore much more sensitive for these scenarios, when being far from critical coupling. The FE extraction method in this form is valid as long as there is no significant additional bending loss in the cavity, i.e. as long as the stronger scattering can be attributed to FE and not additional loss. In that case the additional scattering due to bending would need to be quantified first.

Furthermore, we would like to elaborate why our method is particularly suited for high-Q and off-critical-coupling measurements for the specific example of the used ring resonator. Fig. S7a shows the FE and transmission of a resonance at different coupling regimes. In the zoom-in in Fig. S7b for low FEs, it can be seen that

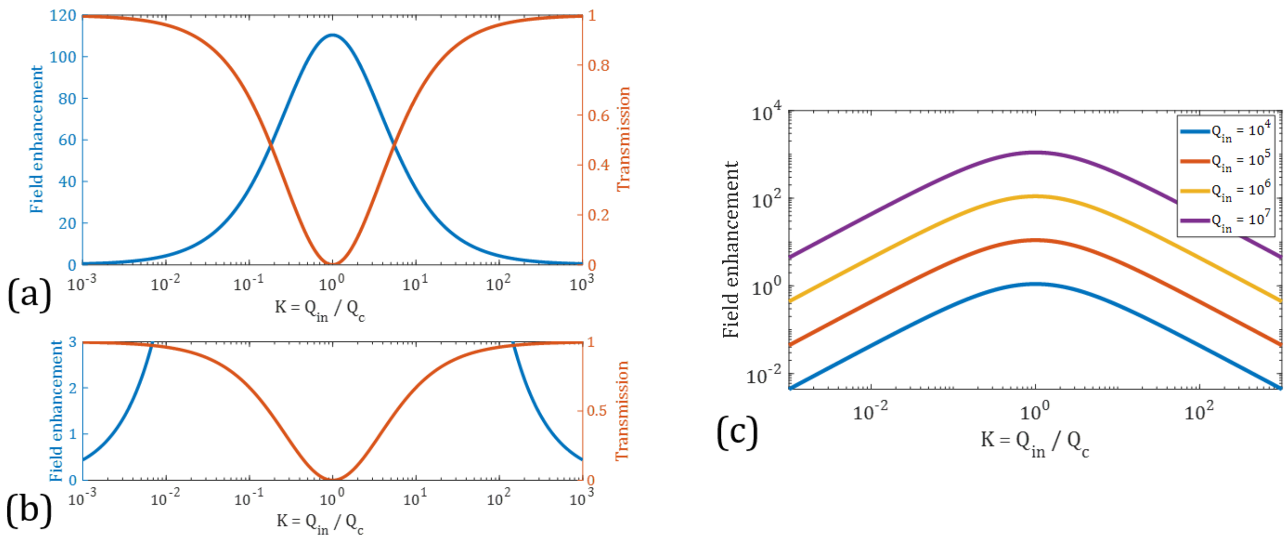

Fig. S7: (a) Field enhancement and transmission of a resonance at different coupling regimes. This is for a SiGe ring with R = 250 µm, $Q_{in} = 10^6$ and λ = 4 µm. (b) Zoom-in on low field enhancements, showing that even for coupling regimes far from critical coupling (more than two orders of magnitude difference between Q_{in} and Q_c) field enhancements of more than 2 can be obtained, which is easily detectable with our method. (c) Field enhancement for different coupling regimes and different intrinsic Q-factors, showing that for higher Q the detection bandwidth of our method increases.

even for coupling regimes far from critical coupling (more than two orders of magnitude difference between Q_{in} and Q_c) FEs of more than 2 can be obtained, which is easily detectable with our method. For the same coupling regimes, there is almost no drop in transmission, meaning that the traditional method would likely miss the resonance. In Fig. S7c we illustrate why our method is particularly suited for high-Q rings. In transmission, the detection bandwidth for different coupling regimes is always the same regardless of Q_{in}. With our scattering method however, the detectable range of coupling regimes is increasing with increasing Q. For example, assuming that the field enhancement should be at least 1 to be easily detectable, i.e. same power in the ring as in the bus, for $Q_{in} = 10^5$, the range of detectable coupling regimes is from K = 0.02 to 50. For $Q_{in} = 10^6$ this already increases to roughly K = 0.002 to 500. In reality, even for the case of lower power in the ring than in the bus, the resonance can be detected until a certain point, so this is just to have an estimate for a lower limit.

Third, we would also like to point out the background and noise considerations inherent to the scattering measurement method. We have a relatively large thermal background as we are working in the MIR. On top of that, there is a non-negligible background signal due to light scattered from other parts of the chip, such as the input chip end-facet for example. This can be also seen in Fig. S6 where we have a background offset of around 5000 off resonance, while the scattering of the ring probed on resonance raises the signal to around 5600. However, the background is largely wavelength independent over the scanning range of the measurement, so it is just a constant offset added to the measured scattered light, which does not affect the resonance spectral signature. The sensitivity of the camera is largely enough to measure devices even very close to the input facet, where background light is the worst (see Fig. 2a+b). The Q factor can then be extracted from the full width at half maximum from the remaining peak, which can also be obtained from fitting a Lorentzian whenever necessary.

As a usual wavelength referencing approach simultaneous to the measurement is limited by the lack of certain commercial MIR components, we perform a different, simple, referencing, where we measure the start and stop wavelength by a wavelength meter and then verify the linearity during the sweep by the regularity of the Fabry-Perot fringes afforded by the chip end-facets. It is ensured that the chip reflection does not introduce any retroaction to the laser source, according to the polarizers that are adequately set on the light path for power control. As reflected by the error bars of the extracted resonance Q-factor values, our wavelength referencing is not the limiting factor for accuracy in the measurement setup and are hence deemed sufficient.

Finally, the measurement method also allows us to estimate the influence of bending losses. As one main contribution to bending losses is the increased interaction of the mode with the sidewall roughness, bending loss would correspond to increased scattering observed with the MIR camera. We validated that with the method we can see bending losses, but for the resonators presented here we did not have any additional scattering due to bending. For example, in Fig. 2b (left) it can be seen that the bending sections in the bus waveguide, which have a bending radius close to the ring, do not show increased scattering. Regarding the propagation loss, it cannot necessarily be assumed that a higher propagation loss will directly lead to a proportional increase in the scattering, as it consists of various contributions. For example, for SiGe waveguides it has been shown in Ref. [2] that the dominating loss contribution was free carrier absorption by residual non-intentional doping, while scattering by sidewall roughness was almost negligible in comparison. While in our waveguides the width is smaller and the confinement factor lower and consequently the effect of the roughness slightly higher, we expect that the general trend is still true for our waveguides.

Simulations

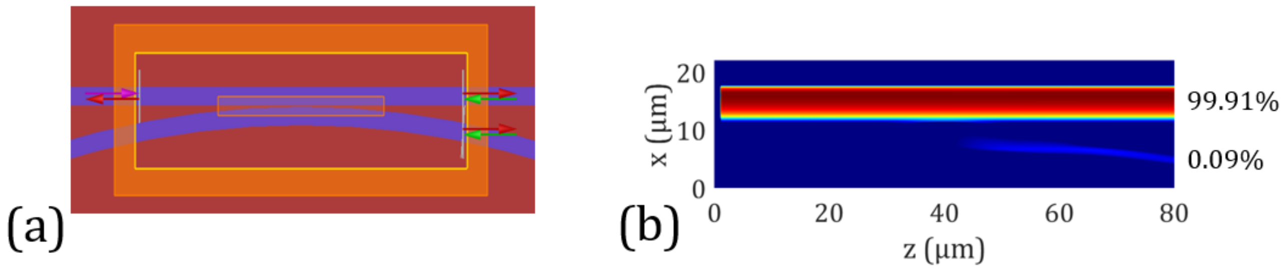

Fig. S8: FDTD simulations for the coupling gap of a SiGe ring resonator (250 µm radius) such as also used in the measurements. In this case the gap is 250 nm, which shows a power coupling of 0.09 %. (a) Simulation setup. (b) Simulated field.

We performed FDTD simulations with Lumerical to simulate the coupling strength at the coupling section. Fig. S8 shows an example of a SiGe ring resonator with a radius of 250 µm and a coupling gap of 250 nm, similar to that used for the bistability measurement. We see that for these parameters, we get a power coupling of 0.09 %, consistent with the measured extinction ratio discussed above. A ring with an intrinsic Q of one million would require a power coupling of 0.90 % to reach critical coupling, so a factor 10 higher. While for a fully etched 250 nm gap, this simulation shows that the rings are still expected to be under-coupled, the situation is expected to be even further imbalanced for the increased gaps of 500 nm that were probed in Fig. 3. Again, we re-emphasize that despite this strong under-coupling regime, our scattering method measurement provides us with a reliable assessment of the high Q rings achievable with our platform. Finally, we note that this strong under-coupling regime, which is undesirable for potential applications like comb generation, can in the future be overcome with more sophisticated coupling strategies such as Pulley couplers for example.

Discussion of Bistability

As we observed optical bistability that can be caused by two different contribution, thermo-optic or Kerr based, we investigate here how each of them could explain the measurements. We have measured the thermo-optic (TO) coefficient for this platform by comparing the free spectral range with the free thermal range from a temperature scan. The result was $\mathrm{dn/dT} = 2.3 \cdot 10^{-4}\ \mathrm{K}^{-1}$, which is consistent with the literature for measurements on Ge and Si individually.[3] The nonlinear index for SiGe has previously been measured by our team as $n_2 = 4.0{\cdot}10^{-18}$ m^2/W.[4] From the bistability measurement, particularly Fig. 4b, it can be seen that the $\mathrm{d}\lambda$ is around 80 pm

(span of the bistable curve minus the width of the cold cavity). This corresponds to roughly $\mathrm{dn}_{eff} = 7 \cdot 10^{-5}$. We calculated a field enhancement factor at critical coupling of around 110 for the resonator, but as we are still quite far from critical coupling, it will be much lower, and the method described above gives an estimate of about 4.5 for this particular ring. Considering that we have 1 W of off-chip pump power, we can assume around 300 mW of power in the bus waveguide. This would give around 1.5 W in the ring. The Kerr effect at this power would lead to a dn_{eff} of $6 \cdot 10^{-7}$ (using $n_2 = dn/dI$ and $dI/dP = 1/A_{eff}$), much less than what we see. If we assume instead that the observed dn_{eff} of $7 \cdot 10^{-5}$ stems from the TO effect, this would correspond to a temperature increase of $\mathrm{dT} = 0.30$ K, which could be a realistic value. To conclude, a small portion of the bistability is likely due to the Kerr effect, but the main contribution has to be from the TO effect to explain the observed bistability.

References

[1] P. H. Wang, K. L. Chiang, and Z. R. Yang, "Study of microcomb threshold power with coupling scaling," *Sci Rep*, vol. 11, no. 1, p. 9935, 2021.

[2] V. Turpaud et al., "Low-loss sige waveguides for mid-infrared photonics fabricated on 200 mm wafers," *Opt Express*, vol. 32, no. 10, pp. 17400–17408, 2024.

[3] B. J. Frey, D. B. Leviton, and T. J. Madison, "Temperature-dependent refractive index of silicon and germanium," in *Optomechanical Technologies for Astronomy*, vol. 6273, pp. 790–799, 2006.

[4] M. Sinobad et al., "Mid-infrared supercontinuum generation in silicon-germanium all-normal dispersion waveguides," *Opt Lett*, vol. 45, no. 18, pp. 5008–5011, 2020.
